# Microstructure and magnetism of austenitic steels in relation to chemical composition, severe plastic deformation, and solution annealing

**DOI:** 10.1038/s41598-025-86028-5

**Published:** 2025-01-15

**Authors:** Kamila Hrabovská, Ondřej Životský, Petra Váňová, Yvonna Jirásková, Lucie Gembalová, Ondřej Hilšer

**Affiliations:** 1 Faculty of Electrical Engineering and Computer Science, VŠB -Technical University of Ostrava, 17. listopadu 2172/15, 708 00 Ostrava-Poruba, Czech Republic; 2 Faculty of Materials Science and Technology, VŠB-Technical University of Ostrava, 17. listopadu 2172/15, 708 00 Ostrava, Czech Republic; 3 Faculty of Mechanical Engineering, VŠB –Technical University of Ostrava, 17. listopadu 2172/15, 708 00 Ostrava-Poruba, Czech Republic

**Keywords:** Stainless steels, microstructure, Magnetic properties, Phase transformation, Magnetic properties and materials, Mechanical engineering

## Abstract

Three types of commercial austenitic stainless steels, 1.4307 (AISI 304 L), 1.4404 (AISI 316 L) 1.4845 (AISI 310 S) with different chemical compositions, are subjected to severe plastic deformation at room temperature by a unique Dual Rolling Equal Channel Extrusion (DRECE) method. Its impact is evaluated from the viewpoint of microstructure analyses, X-ray diffraction, and macroscopic magnetic properties completed by microscopic Mössbauer characteristics. The study also includes the solution annealing at 950 °C for 0.5 h to follow the recovering austenitic structure and paramagnetic state of steels with the aim to offer more information with respect to new technical applications. The results show the importance of the steel’s chemical composition and microstructure, mainly grain size, on the stability of the austenitic structure closely associated with the paramagnetic behaviour.

## Introduction

The growing claims of various industrial branches increase demands on more detailed microstructural and physical properties analyses. This initiates a more sophisticated testing method adjusted to old and new corrosion-resistant materials at their disposal, e.g., drivetrains or electric vehicle components. For example, in the case of at present highly propagated electric vehicles, certain key components such as the protective battery covers, protective housings, and support rings should ideally be made of corrosion-resistant steel being simultaneously paramagnetic. This should minimize the effect of a magnetic field and contribute to an increase of the electric motor efficiency. Among known stainless steels of the ferritic, martensitic, duplex (ferritic-austenitic), and austenitic structures, the only last one is fully paramagnetic contrary to all others being ferromagnetic^1^. However, the properties and performance of austenitic stainless steels are closely related to their microstructures depending on the chemical composition and the cooling rate during solidification^2–4^. Austenitic stainless steels contain 14 to 28%wt. chromium, 2 to 35%wt. nickel, less than 0.15%wt. carbon and other elements such as molybdenum, manganese, copper and others in varying proportions. Their paramagnetic behaviour is characteristic by a low magnetic permeability being about 1.01–1.02. Its increase can be a sign of the undesirable presence of the δ-ferrite and/or a partial diffusion-less martensitic transformation. The amount of induced martensite depends on several factors such as material chemical composition (lower content of nickel, manganese, carbon, and/or nitrogen), plastic strain, strain rate, stress state, grain size, and grain orientation^5–8^. These factors as well as their combinations, e.g., the low temperature, high stress and high magnetic fields have been studied frequently in the recent years^9–11^. The main reasons are new applications of austenitic steels at conditions when the stability of the austenitic structure, paramagnetic behaviour, and other excellent properties of these materials are highly desirable. This is reflected also in many recent studies devoted to chemical composition, microstructure evaluation, and/or, e.g., cryogenic treatment^10–14^.

This work is devoted to detailed study of three commercial austenitic stainless steels, namely 1.4307 (AISI 304 L), 1.4404 (AISI 316 L) and 1.4845 (AISI 310 S). These austenitic steels differ mainly in their contents of Ni, Mo and Cr. In particular, the 1.4845 steel is characterised by highest Cr and Ni contents, while 1.4404 and 1.4307 steels have similar content Cr and 1.4404 steel has Mo addition. The steel 1.4307 features by good weldability, cold ductility, and resistivity up to temperature of 300 °C. The steel 1.4404 can be used up to 550 °C and the last stainless steel, 1.4845, is suitable up to 1100 °C. The present studies were motivated by mostly limited information introduced in the manufacturer’s material sheets. All types of austenitic steels were exposed to cold straining using Dual Rolling Equal Channel Extrusion (DRECE). Detailed changes in the microstructures are followed in their magnetic behaviour. The DRECE method includes Dissimilar Channel Angular Pressing (DECAP) and Continuous Equal-Channel Angular Pressing (ECAP-CONFORM)^15^. It has been developed for forming metal sheets of limited dimension, namely (1000 × 60 × 2) mm^3^.

## Experimental details

### Materials

All austenitic stainless steels, 1.4307 (AISI 304 L), 1.4404 (AISI 316 L), and 1.4845 (AISI 310), were produced by the Argon Oxygen Decarbonisation (AOD) process with additional cold rolling into 2 mm thick metal plate. From these metal plates, the steel strips of dimension 1000 mm in length and 58 mm in width were cut using plasma. All steels in this state are taken as original and the prepared samples are denoted AC. The chemical compositions were checked by glow discharge optical emission spectroscopy (GDOES) using the Spectruma GDA 750 device. The Eltra CS 2000 device was used to determine carbon and sulphur contents by combustion analysis. The obtained results and corresponding values supplied by the producer of stainless steels, both presented in Table 1, show a very good agreement.

The 1.4307 belongs to the most common of all stainless steels similarly as the steel 1.4404 but this steel contains a slightly higher amount of molybdenum promoting higher resistance to acids. The last studied steel 1.4845 belongs to heat-resisting steels, applicable up to 1100 °C, due to a higher chromium content.

**Table 1:** Chemical composition (%wt.) Of studied austenitic stainless steels supplied by producer and obtained by GDOES.

Element	1.4307		1.4404		1.4845	
	Manufacturer’s data	GDOES	Manufacturer’s data	GDOES	Manufacturer’s data	GDOES
C	0.016	0.022	0.022	0.026	0.044	0.040
Cr	17.635	17.91	17.200	16.65	24.550	24.10
Mn	1.637	1.77	1.380	1.66	1.347	1.32
Mo	-	0.23	2.040	2.06	-	0.17
N	0.086	-	0.049		0.028	
Ni	8.015	8.04	10.000	9.83	19.150	18.16
P	0.033	0.032	0.034	0.038	0.028	0.030
S	0.002	0.002	< 0.001	0.001	0.002	0.001
Si	0.361	0.33	0.550	0.51	0.439	0.45
Cu	-	0.29	-	0.34	-	0.21
Ti	-	< 0.001	-	0.001	-	0.003
Co	-	0.148	-	0.203	-	0.151
B	-	0.001	-	0.003	-	0.002
Pb	-	< 0.001	-	< 0.001	-	< 0.001
V	-	< 0.001	-	< 0.001	-	< 0.001
W	-	0.177	-	0.190	-	0.208
Zr	-	0.021	-	0.023	-	0.026
Al	-	0.003	-	0.004	-	0.004
Nb	-	0.028	-	0.023	-	0.023

All steels should embody the austenitic fcc structure and paramagnetic behaviour. The information on welding properties with respect to various types of microstructures provides the WRC diagram^16^ allowing the determination of the nickel and chromium equivalents. The nickel equivalent, Ni_eq_, is calculated using the weight% of austenite stabilising elements and the chromium equivalent, Cr_eq_, using the weight% of martensite stabilising elements. The entering of both these parameters for stainless steel into a diagram according to WRC allows finding the volume content of austenite and/or ferrite in the resulting microstructure. The calculated equivalents, Ni_eq_ and Cr_eq_, including the amount of ferrite content (resp. δ-ferrite), F, are presented in Table 2.

**Table 2:** Calculated values of the nickel, Ni_eq_, and chromium, Cr_eq_, equivalents, content of ferrite, F, and martensitic temperature, Md_30_.

Steel	WRC diagram			
	Ni_eq_ %wt.	Cr_eq_ %wt.	F %wt.	Md_30_ (°C)
1.4307	10.30	17.64	6	13.26
1.4404	11.74	19.24	7	-61.42
1.4845	21.25	24.55	0	-338.89

It is known that the transformation of austenite into martensite is caused by deformation. Parameter Md_30_ in Table 2 expresses that 50% of austenitic phase is transformed into martensite owing to 30% deformation. The low value of Md_30_ presented for the 1.4845 steel characterises the highest resistance to formation of deformation induced martensite and therefore the highest stability of the austenitic structure and thus paramagnetic behaviour.

The samples prepared from all mentioned steels are in their original states denoted AC, those exposed to cold forming by DRECE method by D and after deformation and subsequent annealing AN. The samples were cut and machined according demands of individual experimental methods. The cutting was done using a high-pressure liquid jet to exclude the undesirable structural and chemical changes due to a local thermal heating. The annealing was done in vacuum by a temperature increase of 0.2 °C/s to 950 °C followed by delay of 30 min and a subsequent cooling inside the furnace for about 24 h to the room temperature.

### DRECE method

**Fig. 1:**
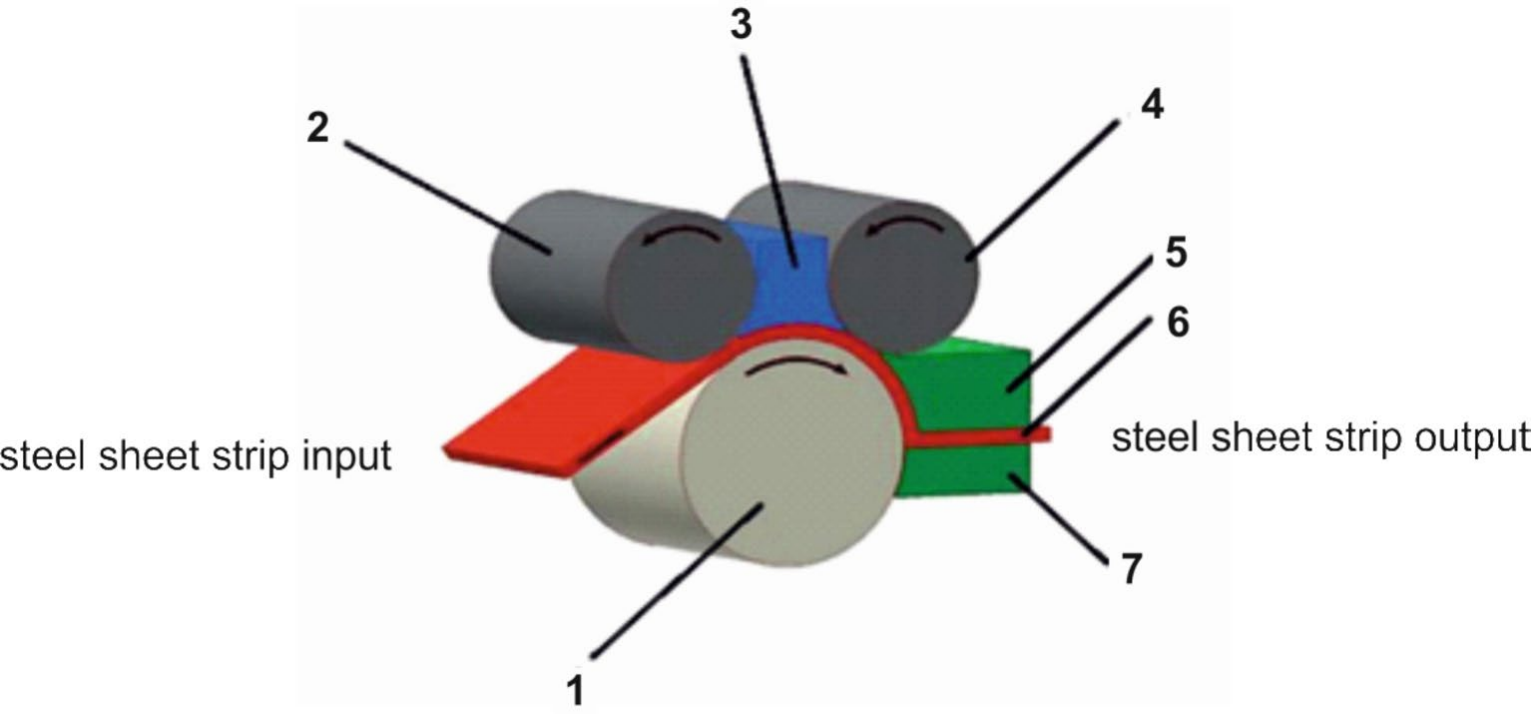
Principle of the DRECE technique for forming the steel sheet strip^17^: 1 – feed roller, 2 – rear pressure roller, 3 – guide intermediate member, 4 – front pressure roller, 5 – upper tool holder, 6 – formed material, 7 – lower tool holder.

A multi-pass rolling at room temperature is an effective cold-forming technique of metal materials contributing to an enhancement of hardness and strength. Generally, the thickness of a rolled material decreases. An advantage of the recently developed Dual Rolling Equal Channel Extrusion (DRECE) technique is that only one pass can be sufficient to improve the mechanical properties of a steel sheet without changing its thickness^17,18^. An extrusion of metal material leads in a deformed zone to the creation of shear stresses initiating an increase of dislocation density, formation of sub-grains, refinement of structure, and thereby increase in hardness. The DRECE technique is schematically shown in Fig. 1.

The DRECE technique was applied for one pass of cold rolling at all studied austenitic stainless steels. This led to significant steel strengthening. The deformation angle α was set to 108°.

**Fig. 2:**
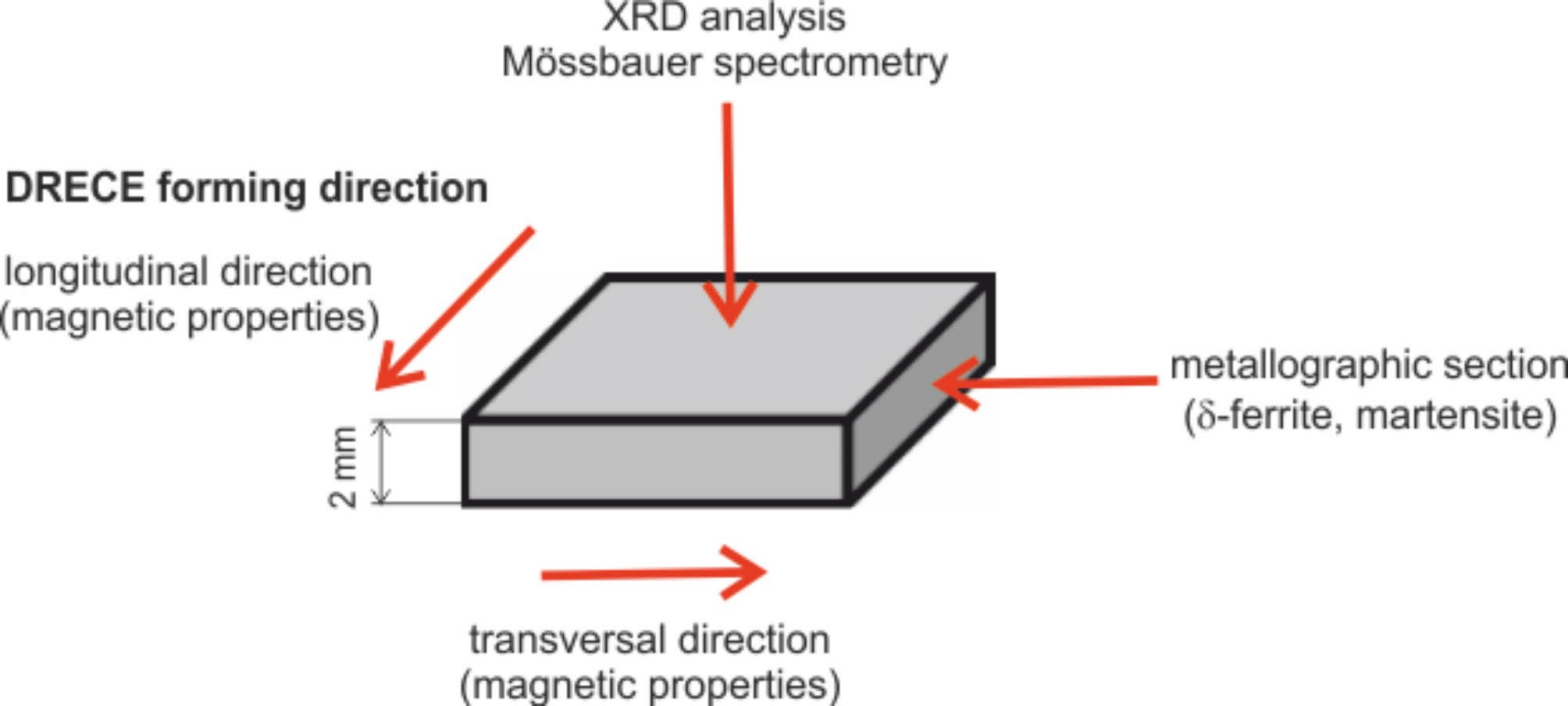
Schematic representation of the steel sample indicating the experiments performed with respect to DRECE cold forming direction.

Figure 2 shows the schema of 2 mm thick steel sample with respect to the DRECE cold forming direction and presents used experimental methods. For magnetic measurements the external magnetic field was applied parallel (longitudinal direction) and perpendicular (transversal direction) to the DRECE. The results of X-ray diffraction and Mössbauer spectrometry were obtained from the upper surface of the sample from a depth corresponding to tens of micrometres. Microstructure analysis for δ-ferrite and martensite evaluation was done on the sample cross-section.

### Experimental methods

#### Microstructure and chemical composition

An Olympus IX70 light metallographic microscope was used to follow the morphology of samples in various states of their preparation and their microstructures were analysed using an image analysis by SW Image Pro Analyzer 7.0. The observations were done on the longitudinal cross-section relative to the direction of the DRECE forming. The sample surfaces were metallographically treated using V2 etchant to make visible the austenitic microstructure and an electrolytic etching in a 10 M NaOH solution at 17 volts for 10 s was used to make visible a prospective presence of δ-ferrite in studied samples. The crystal structure and phase composition of steels were studied using an AXS D8 Advance diffractometer (Bruker, GmbH) equipped with CuKα (λ = 0.1540598 nm) radiation and position-sensitive LynxEye detector. All measurements were realized at room temperature (RT) in 2*θ* range 30°-110° with the step 0.014° and time per step 2 s. Rietveld structure refinement method^19^ and the ICDD PDF-2 database were applied to analyse the measured X-ray diffractograms.

#### Magnetic measurements

The RT magnetic properties were measured using a vibrating sample magnetometer (VSM, EV9, MicroSense) in an applied magnetic field ± 1600 kA/m. The samples in dimension of (5 × 5 × 2) mm^3^ were prepared from steel strips and measured in their original state (AC), after DRECE cold forming (D), and after deformation and annealing (AN).

#### Mössbauer spectrometry

**Fig. 3:**
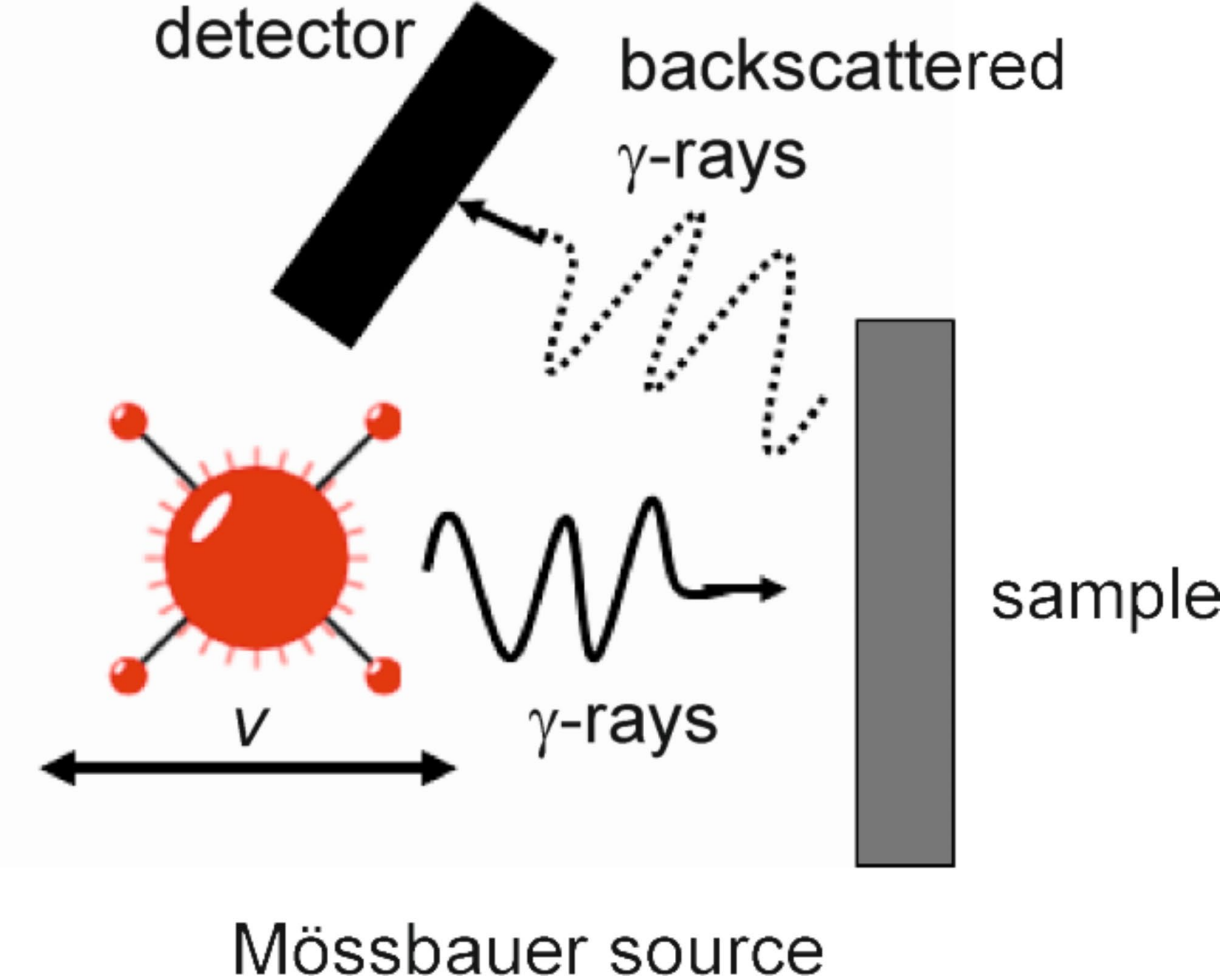
Schema of the Mössbauer experiment in a backscattering geometry.

Home-made Mössbauer spectrometer with a ^57^Co(Rh) source was used for RT measurements of samples using γ-rays in a backscattering geometry schematically shown in Fig. 3. This allows to get information from about 30 μm thick sample surface which can be taken as representative of a magnetic state in a volume of material. More about the Mössbauer method can be found, e.g., in Ref^20^.

The velocity scale calibration was performed with α-Fe and the isomer shifts are given with respect to its RT Mössbauer spectrum. The measurements were done on the samples (20 × 20 × 2) mm^3^ in dimension prepared from the original steel and from the steel sheets after the DRECE forming. All spectra were evaluated within the transmission integral approach using the CONFIT program^21^. In the measured Mössbauer spectrum, the crystalline components are represented by discrete single- (L), double- (DL), and/or six-line (S) Lorentzian sub-spectra determined by discrete values of hyperfine parameters: *δ* - isomer shift(s), *Δ* - quadrupole splitting(s), and *B* - hyperfine induction(s), corresponding to paramagnetic (*δ* and *Δ*) and/or ferromagnetic (*δ*, *Δ*, and *B*) phases, respectively. All components are further described by their intensities, *A*.

## Results and discussion

### Microstructure and phase composition

**Fig. 4:**
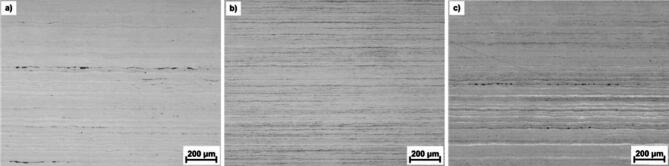
Micrographs in AC state after electrolytic etching in a 10 M NaOH solution for the determination of δ-ferrite for the 1.4307 (**a**), 1.4404 (**b**), and 1.4845 (**c**) steels.

The metallographic investigations of all three steels in the original (AC), cold deformed (D), and deformed/annealed (AN) states were applied predominantly for the detection of the δ-ferrite and/or martensite phases. It is necessary to note that the surfaces of samples are exposed to metallographic treatment important for microstructure visualization as grinding, polishing and etching. The volume fraction of δ-ferrite was determined by image analysis from cross-sections (Fig. 4) in the amount given in Table 3. It can be seen that its fraction in the original AC states of steels 1.4307 and 1.4404 is lower compared to the volume values calculated using the WRC diagram and given in Table 2. However, the contents of δ-ferrite are generally very low in all steels.

**Table 3:** Area fraction of δ-ferrite in %vol. Determined by the metallographic method.

State/steel	1.4307	1.4404	1.4845
AC	0.11	0.46	0.61
D	0.16	0.89	0.27
AN	0.05	0.22	0.15

**Fig. 5:**
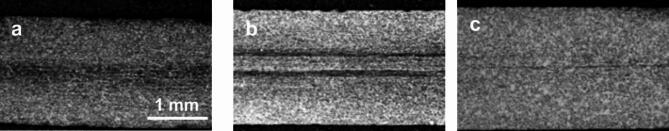
The cross sections prepared in the longitudinal direction with respect to cold rolling using DRECE method for the 1.4307 (**a**), 1.4404 (**b**), and 1.4845 (**c**) steels (etched using V2).

**Fig. 6:**
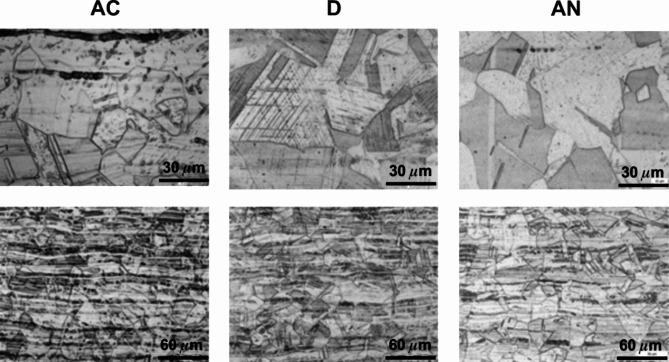
The surface (a, b, c) and centre (d, e, f) microstructure morphologies of the samples prepared from the steel 1.4307 in its original (AC), deformed (D), and annealed (AN) states (etched using V2).

**Fig. 7:**
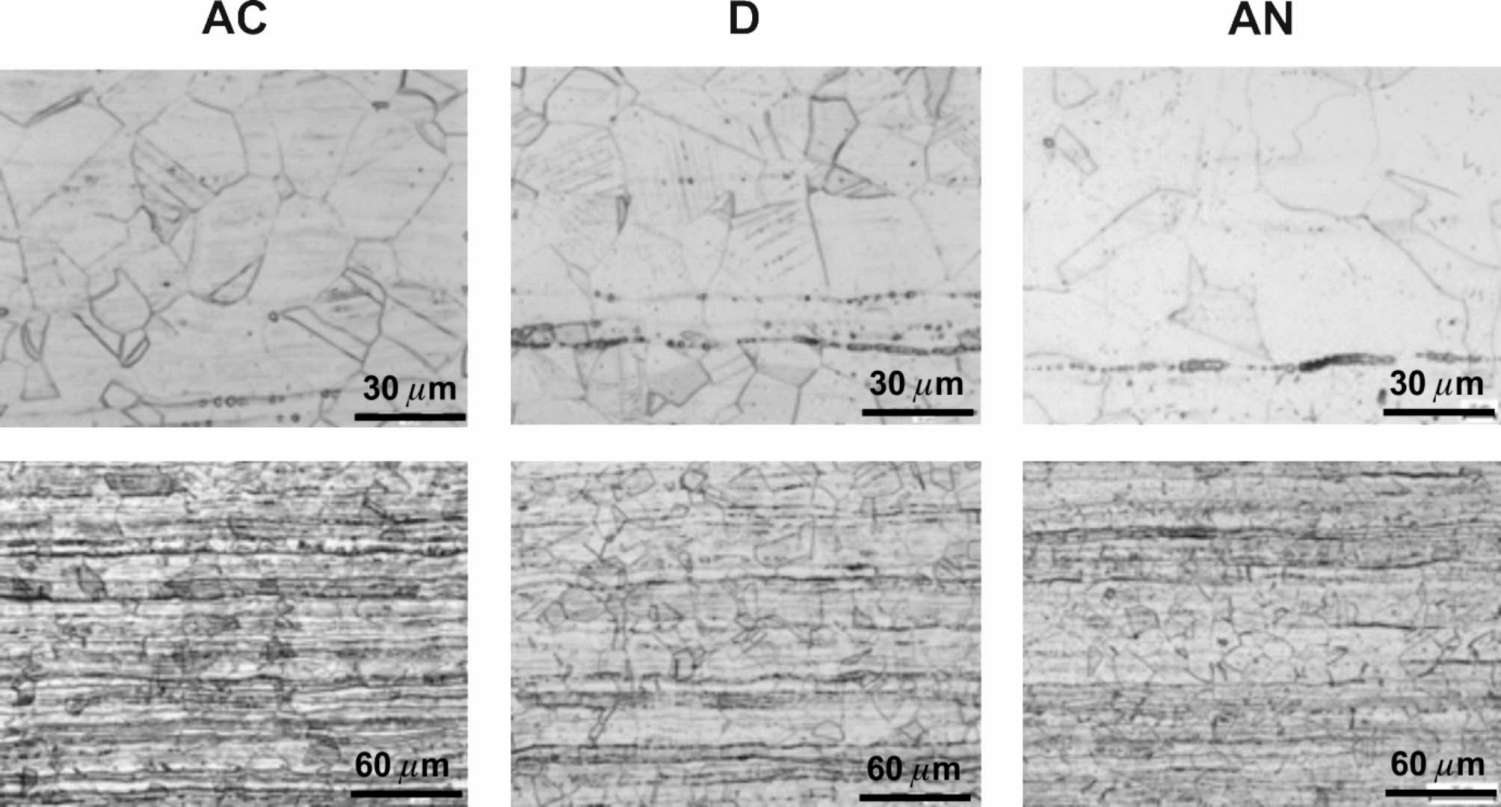
The surface (a, b, c) and centre (d, e, f) microstructure morphologies of the samples prepared from the steel 1.4404 in its original (AC), deformed (D), and annealed (AN) states (etched using V2).

**Fig. 8:**
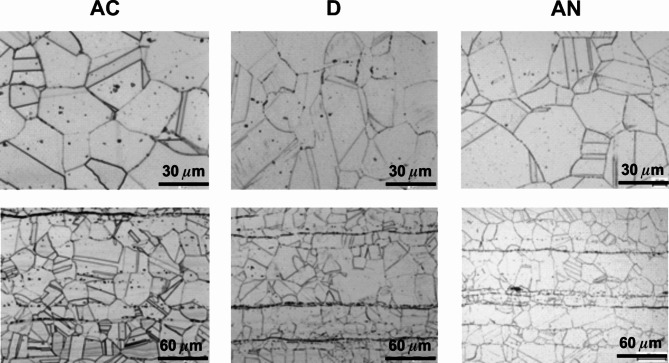
The surface (a, b, c) and centre (d, e, f) microstructure morphologies of the samples prepared from the steel 1.4845 in its original (AC), deformed (D), and annealed (AN) states (etched using V2).

The microstructure, initially observed on the cross-sections prepared from the steels after cold rolling in the longitudinal directions (D) in Fig. 5, discovers somewhat different microstructures at the surface compared to the bulk of all steel samples. This was subsequently confirmed by more detailed studies presented for the samples in all, AC, D, AN, states in Figs. 6, 7 and 8.

Figure 6 shows the most common austenitic steel 1.4307 whereas the upper panel is devoted to the surface and the bottom panel to the bulk microstructures. The mean grain size of 30 μm was determined for both AC and D states while the annealing has contributed to its slight increase to about (40–50) µm. The microstructure in the centre of the cross-section is strongly affected by deformation after cold forming to a thickness of 2 mm. In the centre, dense rows containing δ-ferrite and deformation-induced martensite formed by diffusionless transformation of austenite occur. The same transformation is more pronounced on the surface of the sample in state D, where bands of deformation-induced martensite occur in individual grains. Nevertheless, the subsequent annealing has contributed to the elimination of these undesirable phases as seen in the AN state.

The surface and bulk microstructures concerning the austenitic 1.4404 steel are shown in Fig. 7 in a similar way as presented above. It is also the common austenitic steel slightly enriched in molybdenum content. A comparison of both steels from the viewpoint of the surface and bulk microstructures does not reveal any substantial differences (see Figs. 6 and 7). The mean grain size of the 1.4404 steel in its original AC state ranges between 20 μm and 30 μm without any marked changes in the D and AN states. The elongated narrow particles observed in its original AC state near the surface are oriented towards the centre of a sheet thickness and represent a small amount of δ-ferrite (Fig. 7, AC). Moreover, the deformation bands in the bulk of this sample (Fig. 7, D), less pronounced in comparison to the deformed 1.4307 sample, originate also from the diffusionless transformation of austenite into martensite.

The results concerning all states of the sample prepared from the 1.4845 steel are presented in Fig. 8. The average grain size of 30 μm in the original AC state remains unchanged after cold forming, D, and only slightly increases to about (40–50) µm after annealing (AN). On the other hand, the somewhat thicker grain boundaries can be seen. The δ-ferrite at the surface of the AC sample (0.61%wt., Table 3,) is also detected by microscopic observations in the bulk in the individual rows together with weakly visible deformation bands. It is inconsistent with the calculated zero bulk value in Table 2. As it concerns the D sample, the deformation-induced martensite is detected at the surface and the individual rows of δ-ferrite are seen in the centre of the sheet thickness. The annealing did not contribute to any substantial changes in the microstructure and phases present (Fig. 8, AN).

**Fig. 9:**
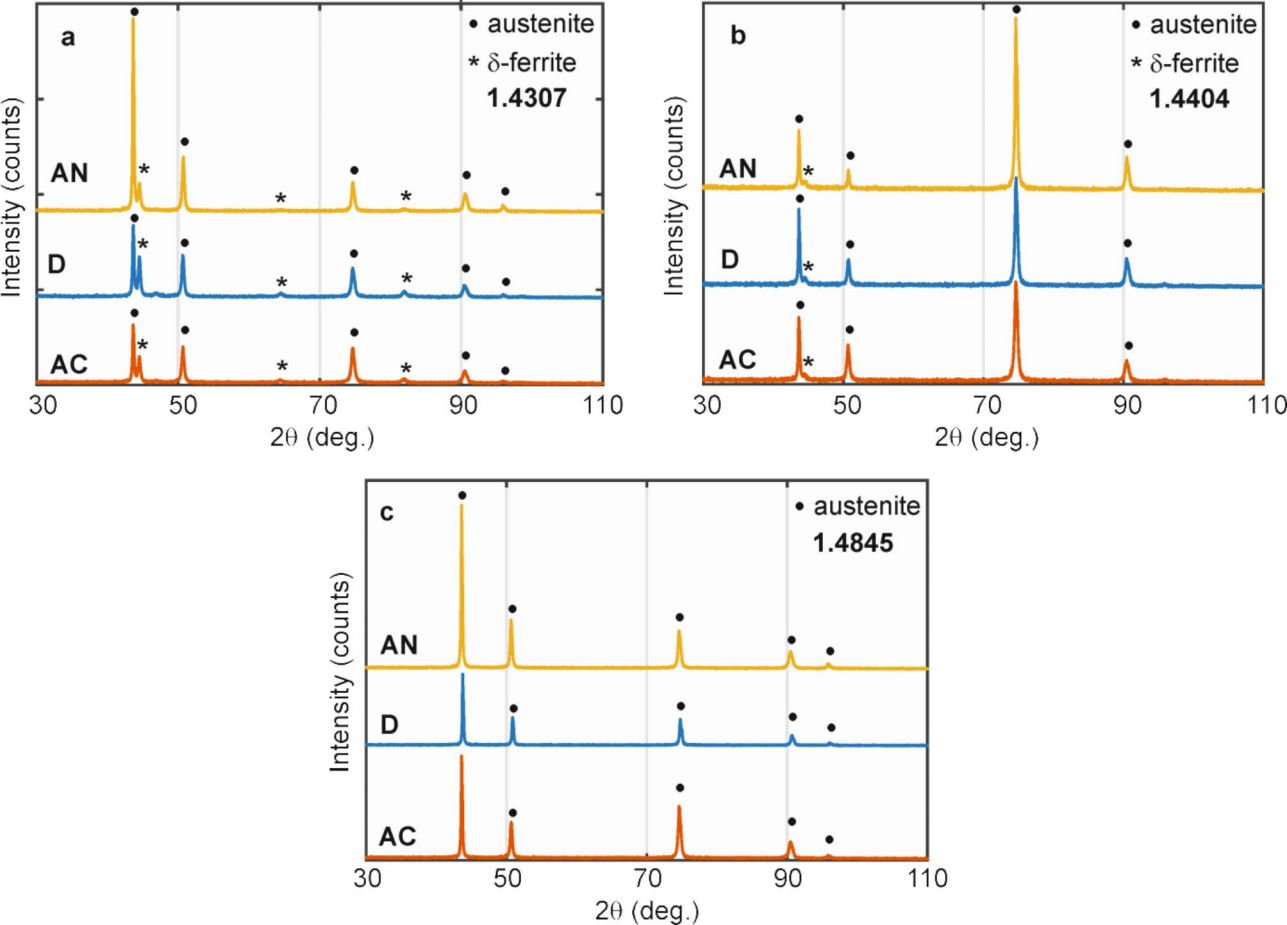
Room temperature XRD patterns of 1.4307 (**a**), 1.4404 (**b**), and 1.4845 (**c**) steels in original (AC), deformed (D), and annealed (AN) states.

The morphology analyses are well supported by the XRD measurements. The diffractograms obtained from the surface layers of approximately 10 μm in thickness are shown in Fig. 9 and the results of their analysis summarised in Table 4. They confirm a dominant amount of austenite phase in all investigated steels. Nevertheless, the only austenite is determined from the diffraction patterns of the 1.4845 steel in all investigated states (AC, D, and AN). About 0.5%wt. of δ-ferrite is present next to austenite in the AC state of the 1.4404 steel. This amount has increased to 1.1%wt. after deformation (D) without any important change after annealing as it is documented in Table 4. The highest content of δ-ferrite of 10%wt. was present in the AC state of the 1.4307 steel. It has increased to about 14%wt. after deformation (D) and decreased to about 10%wt. after annealing (AN). But it is important to note that it is not possible to distinguish δ-ferrite from martensite using XRD analyses. The increase in structural components with a bcc lattice in the deformed state must be due to martensite formation and not to an increase in delta ferrite.

**Table 4:** The results of Rietveld analysis of steels in original (AC), deformed (D), and annealed (AN) states; lattice parameter (*a*), phase content (*I*).

Steel / state		*a* (nm)		*I* (%wt.)	
		Austenite	δ-ferrite	Austenite	δ-ferrite
1.4307	AC	0.3592(9)	0.2878(1)	89.7	10.3
	D	0.3594(4)	0.2878(2)	85.6	14.4
	AN	0.3591(2)	0.2874(9)	90.0	10.0
1.4404	AC	0.3594(1)	0.2880(8)	99.5	0.5
	D	0.3592(4)	0.2875(3)	98.9	1.1
	AN	0.3592(8)	0.2875(9)	99.1	0.9
1.4845	AC	0.3594(3)	-	100.0	-
	D	0.3585(7)	-	100.0	-
	AN	0.3594(4)	-	100.0	-

### Magnetic properties

**Fig. 10:**
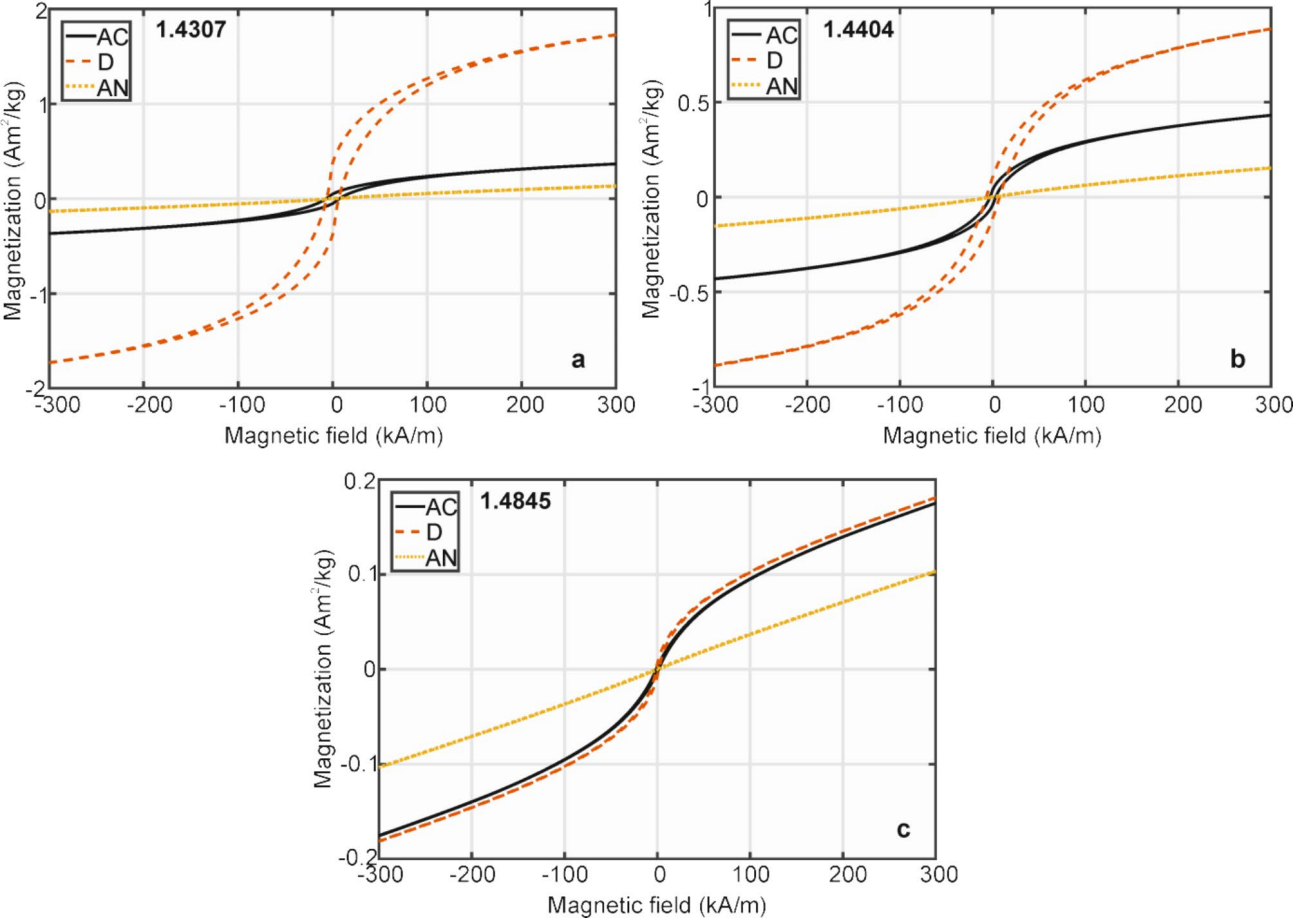
Magnetization curves in the longitudinal direction and the applied magnetic fields ± 300 kA/m for the 1.4307 (**a**), 1.4404 (**b**), and 1.4845 (**c**) steels in the original AC, deformed D, and annealed AN states.

The RT magnetic measurements of samples in their all states (AC, D, AN) were done by applying an external magnetic field *H* (± 1600 kA/m) along the longitudinal and transversal directions. Nearly all measurements have resulted in a superposition of linear magnetization curves in the higher applied magnetic fields and hysteresis curves approximately in the range of magnetic fields ± 300 kA/m. The linear part of the curves reflects the dominant contribution of a paramagnetic phase while the hysteresis curve is characteristic of the ferromagnetic behaviour of a material. Because the measurements in the longitudinal and transversal directions were practically identical, only the curves measured in the longitudinal direction are presented. The details of magnetization curves in the applied magnetic field ± 300 kA/m are shown in Fig. 10 for all steels in their AC, D, and AN states. The magnetic characteristics, coercive fields, *H*_c_, and remnant magnetization, *M*_r_, determined from the hysteresis curves are summarized in Table 5 together with magnetization *M*_2_ determined at magnetic field 1600 kA/m (~ 2 T).

For a pure full-paramagnetic material, the values of *M*_2_, *M*_*r*_, *M*_*r*_/*M*_2_, and *H*_*c*_ should be very low. From this viewpoint, the lowest values of the coercive field and *M*_*r*_/*M*_2_ ratio obtained for the 1.4845 steel in its AC state evidence the dominating paramagnetic austenite. Moreover, its stability is confirmed by the similarly low values of magnetic parameters determined after deformation. On the other hand, the magnetic characteristics presented in Table 5 for steel 1.4307 reflect the lowest resistance to deformation-induced martensitic transformation. It is clearly demonstrated by a dominant hysteresis curve representing martensite next to the minor linear magnetization curve of the paramagnetic austenite. Nevertheless, the magnetic measurements of all steels in the annealed states, AN, have resulted in the linear dependences of magnetization in the whole range of applied magnetic fields (see Fig. 10) confirming a recovery of paramagnetic austenitic structure in agreement with conclusions of the microstructures observations.

**Table 5:** Magnetic parameters of steels in the original, AC, and deformed, D, states obtained from the magnetization curves measured in the longitudinal and transversal directions; *M*_2_ – magnetization at a magnetic field 1600 kA/m (2T), *M*_*r*_ – remnant magnetization, *H*_*c*_ – coercive field.

Direction of magnetic field	Steel		M_2_ Am^2^/kg	M_*r*_ Am^2^/kg	M_*r*_/M_2_ -	H_c_ kA/m
Longitudinal	1.4307	AC	0.803	0.049	0.061	6.855
		D	2.383	0.376	0.157	6.343
	1.4404	AC	0.897	0.038	0.042	3.023
		D	1.430	0.106	0.074	7.101
	1.4845	AC	0.594	0.003	0.005	1.424
		D	0.600	0.006	0.010	1.395
Transversal	1.4307	AC	0.742	0.046	0.062	6.326
		D	2.521	0.640	0.253	9.071
	1.4404	AC	0.840	0.038	0.045	2.864
		D	1.203	0.081	0.067	6.803
	1.4845	AC	0.522	0.007	0.013	0.279
		D	0.557	0.007	0.012	0.159

### Mössbauer spectrometry

The high sensitivity of Mössbauer spectrometry to magnetic behaviour of materials is its high benefit in magnetic phase analysis and in detection of phases formed after different thermal or plastic deformations. For example, Blachowski and coworkers^22^ studied duplex steel in a form of foils and powders influenced by plastic deformation or Bilmes and coworkers^23^ have detected paramagnetic austenite particles in the ferromagnetic martensitic steel associated with transformation-induced plasticity (TRIP).

In the present study the Mössbauer spectrometry was used to confirm paramagnetic state of studied austenitic stainless steels and, eventually, to detect a presence of a ferromagnetic phase(s) with respect to mechanical treatment of samples. The austenite is a paramagnetic phase in which only the electric monopole (isomer shift, *δ*) and electric quadrupole interactions (quadrupole splitting, *Δ*), in dependence on chemical composition and atom ordering, are present. They are represented in Mössbauer spectrum by single line (L) and double lines (DL) components, respectively. Contrary, the ferrite or martensite are ferromagnetic phases in which magnetic dipole interactions are manifested in Mössbauer spectrum by six lines component (S) characterised moreover by hyperfine magnetic field (*B*).

**Fig. 11:**
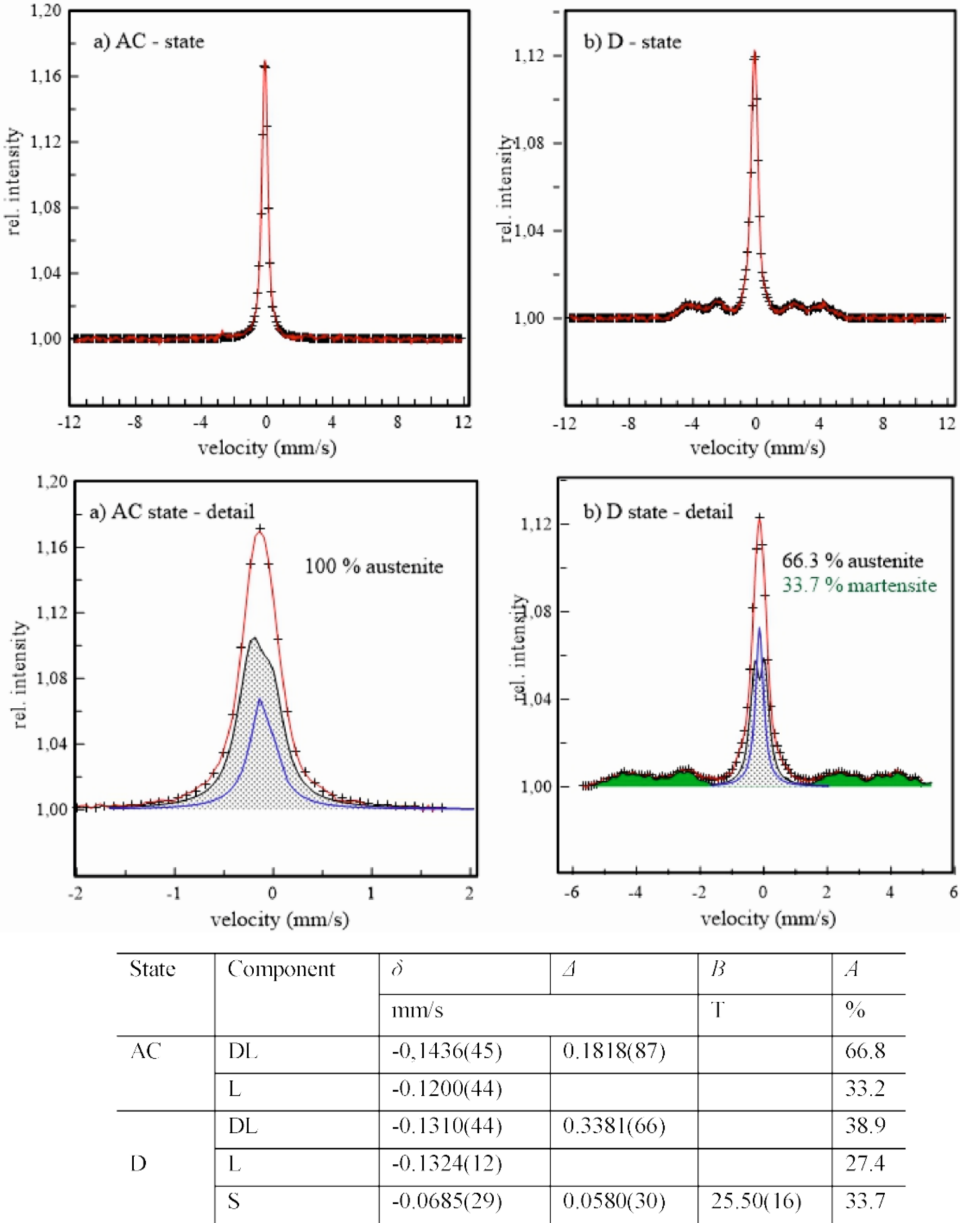
Mössbauer spectra of the 1.4307 steel in its original (**a**) and deformed (**b**) states - upper panel; corresponding subcomponents in experimental spectra - bottom panel; table of hyperfine parameters: isomer shift *δ*, quadrupole splitting *Δ*, hyperfine magnetic field *B*, and intensity *A*.

**Fig. 12:**
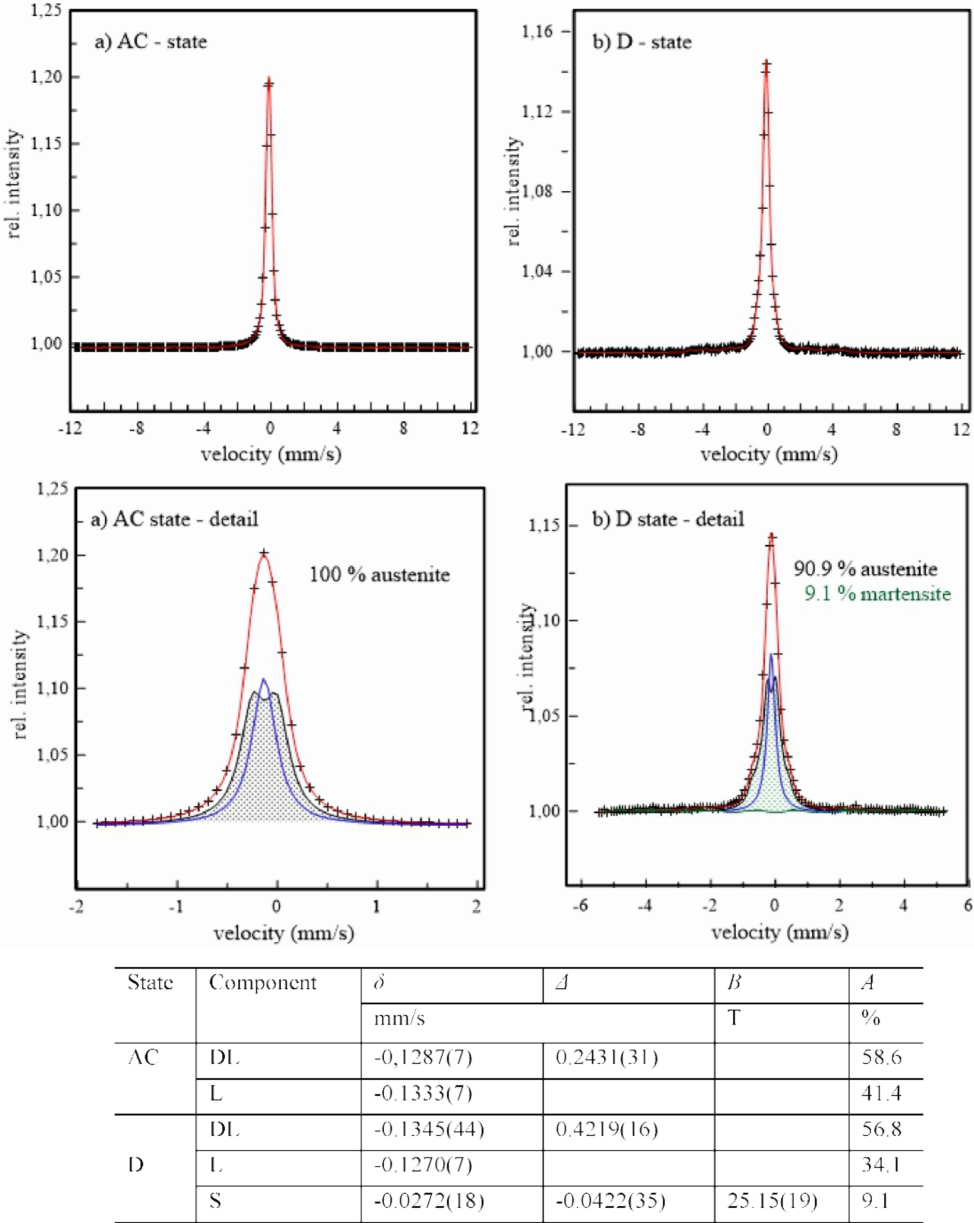
Mössbauer spectra of the 1.4404 steel in its original (a) and deformed (b) states - upper panel; corresponding subcomponents in experimental spectra - bottom panel; table of hyperfine parameters: isomer shift *δ*, quadrupole splitting *Δ*, hyperfine magnetic field *B*, and intensity *A*.

**Fig. 13:**
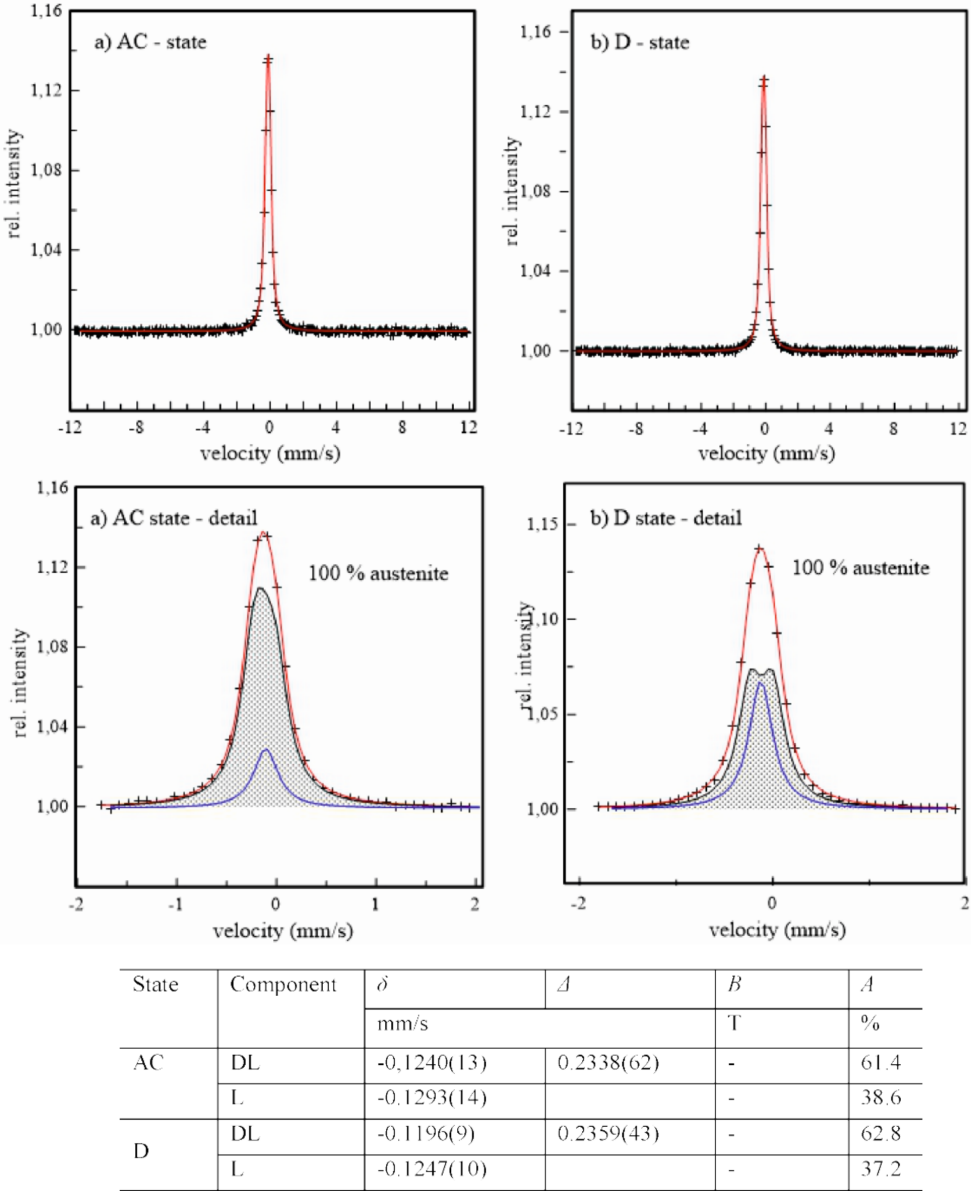
Mössbauer spectra of the 1.4845 steel in its original (**a**) and deformed (**b**) states - upper panel; corresponding subcomponents in experimental spectra - bottom panel; table of hyperfine parameters: isomer shift *δ*, quadrupole splitting *Δ*, hyperfine magnetic field *B*, and intensity *A*.

Representation of steels, 1.4037, 1.4404, and 1.4845 in their original, AC, and deformed, D, states by Mössbauer spectrometry in Figs. 11 and 12, and 13 consist of experimental points (+) and their analysis (full line). The panels above show the measured spectra in the whole range of velocity scale corresponding to the maximal possible value of the hyperfine magnetic field. The panels below represent a part of measured experimental spectra and corresponding sub-spectra formed either with one L, DL, and/or S subcomponents or with sum of these subcomponents represented by the mean values of hyperfine parameters in tables below.

The presence of only single- and double-lines sub-components in the spectra of all steels in their original, AC, states confirm the presence of only paramagnetic austenitic phase. No magnetically split component, corresponding to δ-ferrite, is observed. This confirms its very low content being below sensitivity of the present Mössbauer measurements. After deformation by DRECE technique, the six-line sub-spectra are clearly seen in samples 1.4307 and 1.4404. Nevertheless, they reflect a presence of the ferromagnetic martensite phase with mean hyperfine magnetic field slightly above 25 T. Its content is markedly higher at the 1.4307 steel, 33.7 wt%, in comparison to 9.1 wt% at the 1.4404 steel. No ferromagnetic phase is present in the 1.4845 steel reflecting its good mechanical stability and thereby resistance of austenite against martensitic transformation supporting previous results. The high austenite stability of steel 1.4845 presented in this study is primarily influenced by the higher Ni content. The smaller grain size may also contribute to the higher austenite stability, as presented in the work of H. Mirzadeh and A. Najafizadeh in Ref. ^24^. However, this hypothesis would need to be confirmed by further measurements for the studied steels.

## Conclusions

The present detailed physical studies offer more information concerning commercial austenitic stainless steels, 1.4307, 1.4404, and 1.4845 compared to data introduced in the manufacturer’s material sheets. The steels in their original states are subsequently exposed to severe plastic deformation using the relatively new DRECE method and finely annealed. The stability of the austenitic structure and paramagnetic behaviour are followed in all states with respect to chemical composition, grain size, and phase composition closely connected with the presence of δ-ferrite and the resistance to martensitic transformation. The obtained results can be summarized subsequently:

1. The calculation of martensitic temperature Md_30_ has indicated the highest stability of the austenitic structure for the 1.4845 steel. The experimental results of the microstructure, X-ray diffraction, magnetic measurements, and Mössbauer phase analysis has confirmed austenitic structure in all states and thereby the best stability of paramagnetic behaviour.
2. The other two investigated steels, namely 1.4307 and 1.4404, are not substantially different from the viewpoint of their surface and bulk microstructures. Somewhat different situation is in sensitivity to deformation evoking the martensitic transformation. From this viewpoint the lowest resistance was found to be at the 1.4307 steel embodying the content of martensite after deformation by DRECE technique nearly 34%, while the steel 1.4404 about 9%. Both values were determined using Mössbauer phase analysis being in good agreement with the results of the X-ray diffraction and magnetic measurements. Nevertheless, the annealing has contributed to reverting the martensitic transformation, recovery and re-crystalising the austenitic structure and thereby observing a fully paramagnetic behaviour.
3. From the viewpoint of technical applications where the stability of the paramagnetic behaviour of the austenitic steel is highly desirable, the chemical composition and microstructure, mainly grain size, are both important factors. The steel 1.4845 embodied the highest Ni and Cr contents according to the manufacturer’s material sheet and GDOES analysis and simultaneously the smallest grain size compared to the other, 1.4307 and 1.4404, investigated austenitic steels.

## Data Availability

The data that support the findings of this study are available from the corresponding author upon reasonable request.
